# Essay on Professional Fees

**Published:** 1852-10

**Authors:** Elisha Townsend


### ARTICLE II.

*Essay on Professional Fees.* By ELISHA TOWNSEND, M. D., D. D. S., *read before the American Society of Dental Surgeons, at the Thirteenth Annual Meeting, held at Newport, August,* 1852.

MR. PRESIDENT AND GENTLEMEN:—At your last annual meeting, held in Philadelphia, you did me the honor to invite me to prepare and read before this association, an essay on some subject connected with the Theory or Practice of Dental Surgery, at its present meeting. In answer to this call I appear before you, and will endeavor to give you such reflections as I have, upon a subject intimately connected with the practice of dental surgery, and having, *perhaps,* a greater bearing upon its respectability, and the worth and talent to be employed in its future cultivation, than may at first sight be imagined. I have chosen for the subject of my reflections professional *fees,* and *hope* it "will pay" for the time and attention required. The word fee is properly used to express the compensation, reward, or acknowledgment of services of the more honorable kinds, in contradistinction to those which are merely laborious, and whose motive equivalent are alike expressed by the word *wages.* The usages of language make nice, but suggestive, distinctions, which will serve for useful hints in the matter in hand; thus we speak of the *price* of commodities, the *wages* of labor, the *hire* of servants, the *salaries* or stipends of governors, judges, settled clergymen and teachers, when their recompense is paid for fixed periods by previous stipulations, and limited by former contracts. But the freer and more voluntary remunerations of physicians and lawyers is always called a *fee,* or *honorarium,* because, besides the idea of compensation, it contains the other idea, of being as a token of respect, and not as a complete equivalent. Indeed the primary meaning of the term was rather that of a gratuity, or a cordial acknowledgment for liberal and noble services. The Roman patron actually received no money reward for his services to his client, and to this day, an action at law cannot be maintained in England, for the fee of a physician or counsellor, so strong seems to have been the feeling that the exertion of talent and skill without measure; the dedication of a life to an arduous and responsible profession, and the sympathies and confidences which bind the parties together, should be wholly honorary, both in the benefits confered and their substantial acknowledgment. And there is at least this much reason in the rule, that as the law cannot proscribe or *exact* the excellence of the services expected, so it will not attempt to enforce the gratitude deserved. But whether this refinement has given way before the ruder necessities of business for *good* reasons or not, it is nevertheless obvious, that a professional fee has in it something more than an equitable return for labor and time, something of an appreciation tribute to talent, skill, and care, which cannot be the subjects of contract, and do not admit of a market value estimate. I do not intend to say that men should be governed exclusively by their highest motives, for they have many necessities, but only that all should be respected, and the noblest should form the standard. Some of our interests push us into action without our will, some invite in accordance with our tastes, and, the public has requirements besides, which we must honorably meet. It is happily true, also, that every business necessary to the individual has, in its degree, some worthy connection with the world's wants, and welfare, which give occasions for the union of noble and generous sentiments with the pursuit of private benefits. There are two parties always immediately concerned in professional relations, the individual and his assortments of clients or patients, and a third is never lost sight of by high minded men, that abstract and impersonal, but very real party which we call the world, society or humanity; each of these has proper claims to be provided for and duly considered, and there is no department of professional life and duty which has not its bearings upon them all; even the matter of fees will be found upon full examination to rank high in the policy of professional conduct, notwithstanding the mere mercenary element; when we endeavor to bias a system of compensation upon principles of even justice to all parties, and wholesome influences upon all the interests involved. There is nothing absolute or intrinsic, it must be remembered, in price or value, however essential or ordinary the subject may be; especially, there are some things that are never sold, and there are some others for which no adequate return can ever be made. Commodities are valued at the cost of their production, modified by demand and competition, but services admit of no positive rule. The lower kinds of labor are sold and exchanged for such price as is supposed to compensate the physical toil that is required; bones and muscle need nothing but food and raiment, and for their use, their sustenance and comfort is, perhaps, a fair equivalent; but as we ascend among the imponderables, which the arts and sciences bring into the market, along with the material which they employ, weight and measure, cost and worth, grow more and more out of the reach of commercial arithmetic. Stockbrokers and merchants, though much given to speculation, would be puzzled to fix a rule for the appraisement of the statues of Stuirhauser, the songs of Jenny Lind, or the pictures of any one of the old masters, and it is even more emphatically true, that where extraordinary talent and high training are combined with the best moral qualities in the practice of a liberal profession, nothing which the recipient can pay, in any wise measure the value of that which he receives, whether we estimate it by the use it serves, or, by the qualities of head and heart which it requires. Such is the verdict of the patients themselves who are best able to judge, though they are also most interested in a moderate valuation. Indeed, it may be stated as a rule, that low fees are paid with the least cordiality, because they are of the most ordinary grades of benefit, while high fees bring along with them the liveliest emotions of appreciative regard, for they are the acknowledgment of highest services and most valued reliefs. The avocations which require high talent and skill, imply the cost of previous education and practice, and all the dedication of time and denial of self which eminent attainments demand. There is a condition of labor in which the man and all his faculties are surrendered to the masterdom of another, but, if such surrender of self and life and powers to the advantage of others were the only idea in the word slave, the artist, scholar and man of science would deserve it much more absolutely, for his abnegation is as entire, and the faculties offered in sacrifice are infinitely greater, and the zeal of his own enthusiasm fires the gift upon its altar. His devotion knows no limits; his services no holidays, and if he receives the means of luxurious living from those he serves, it is most generally at the expense of relinquishing the opportunities of their enjoyment. It is in some perception of these hard conditions, that the public accords to professional men, rates of remuneration which compare so largely with the wages of ordinary labor. The thought is, that they should have the means of such accumulation as will at some early day give them their chance of individual life, so well earned, and for whose enjoyment their capacities are so highly cultivated. Besides, there is a tacit expectation that in any position they may attain, they will be taxed with the toil and expense of that general progress of society which they are so well fitted, and so well disposed to promote. Whether the entire philosophy of the principle is compassed or not, there is always at least an instinctive feeling, even in the least reflecting, that large rewards to eminent talents are completely justified. People *may* higgle about the price of commodities in the market place, but they are generally too wise to purchase *cheap talents* when *great* interests are at stake. Moreover, the *un*professional man needs an *in*surance as much as he needs the reliefs and services, and he is willing to give as much or more for *that*, as for the substance of the service itself; nor is such an investment either improvident or unwise. Such reflections suffice to show that there really is no intrinsic limit to the price of professional service, and no external standard by which its absolute value can be measured; yet it has its limitations in fact, and these are found in considerations which are consistent with its honor, and attentive to its necessities, while they restrain the justice of its demands; these considerations are, the fair and comfortable ability of the client or patient to afford a living remuneration for the benefit he receives. He pays not what he owes, but what he can, and what the operator needs, according to the usual average of their respective conditions in society. There is no other rule than that of such accommodation, for there is no principle of price in the transaction. Hence the usage of large fees from the wealthy, and less from the less opulent, and gratis services to those who are quite unable to meet the lowest rates which it is creditable to receive; this doctrine also makes room for those in every profession who address themselves purposely to practice less liberally remuneration than the highest grade, for the whole matter of fees stands upon a sliding scale of compensating adaptations, and the true principles of charge make safe and convenient provision for every sphere of practice; with this difference, be it observed, that the nearer the summit of the scale, the more nearly are all the highest conditions and requirements of duty met and provided for; on the lower side of the medium all the danger lies, above it are all the best securities for all the interests of all parties concerned. Still no principle is violated while the lowest limits of respectability are avoided.

In that sort of labor which requires but little natural skill, and *no* learning, underworking is destructive to competitors, for price is the only point in which their rivalry has play, and abatements *there* work inavoidable mischief, and strikes, and violence against offenders are the *only* remedies, however ineffectual they may be. It is not so in occupations where skill and education have an infinite power of making and marking distinctions among the candidates for business. The better qualified are relieved, in great measure, from the mischief of undercharging by others, except in those departments of their calling which are either really or apparently easiest of execution. The better qualified men are injured, indeed, by such improprieties in their "*esprit du corps*," their common profession is deteriorated, and the public is wronged, but they suffer no special individual injury, and may, therefore, be regarded as wholly unselfish in any solicitudes they may have for reform. With the unreflecting and incapable they will not succeed, but such loss they can well afford, their resource is in still higher and higher attainments, and correspondingly higher charges, and the path of duty and honor happily points in the same direction. The quarrel about low prices may be left in the hands of those who are contented with mere respectability in their profession, and those who are desirous to sustain its dignity, and secure their own ease merely on the ground of dignity, without its proper *excelsior* spirit of achievement.

If any one says, "I am undersold by my neighbor, in the same business," the answer is, bring to market products, which no man can sell for less than your demand, and your complaint is cured. If it is wares or work only, that you have for sale, you must meet competition by cheapening the cost of production, but if it is talent and the attainments of study and discipline, then make them richer and dearer, and your success is certain. This is the natural reward of progress, and the appointed discipline to effect it.

I have, therefore, nothing to say against low charges except what stands of itself as the *per contra* in the accounts I would open to the credit of high rates. To which, for a moment, I now ask your attention, and trust the inferences to make good whatever complaint really lies against the opposite policy, procedure.

First liberal rates of professional fees are justified against the charge of extortion, for no wrong is done to the patient so long as the opportunity of choice is left freely open to him. In our profession this is always the case, and the two parties to the business bargain are both equally in condition to take care of themselves and their own interests, and may, therefore, be trusted with them, without appeal to any other body's conscience or opinions. I do not say, however, that the possessor of a rare excellency, or a happy discovery in his art, may hold its benefits at a *killing* price; but we can, nevertheless, leave him to his discretion, if he happens to be insensible to liberal feelings, for the world will manage to get along without the man who is unwilling to serve it on conscionable terms, as it did before he or his discoveries were known, but thanks to the mutual dependency of all men, we need not fear so hard a bargain as this, nor provide against it, for the individual's interest is concerned in a ready acceptance of what he has to offer to the world; and so there are always two parties to the bargain to keep the balance level. Fees are thus secured against excess, and they are still further limited by the competition of equal ability in the same vocation, and if every thing else failed, they would be held down by that sufficiently resolute self-interest, which in every thing, resists exorbitancy; and thus we may dismiss this aspect of the question, and turn to that view of it which warrants and enjoins a high standard of remuneration.

In the first place, our profession notoriously needs the general character which fair self appreciation may help to give it. It is but one branch; and as yet, the only branch of surgical art which is separated from the others in practice, for this reason, it is held in the *public* opinion to be the simplest of them, and separated for that simplicity, and its supposed remoteness from the general healing art. The better thinkers see in this the fact, that it is only the first to give to itself a proper distinctness of culture, and the opportunity of such distinguished advancement as concentration upon specialty is known to confer upon all the pursuits of men which have much importance and complexity; in the judgment of the most capable, therefore, it is in no danger of such misconstruction. But it remains for the faculty to take care of its rank and standing with the general public, by charging up to the level of its real professional dignity, that the science, skill and responsibility in it, may be felt in all the claims which it justly advances for popular estimation.

*Second*—On the sheer ground of pecuniary equity. The remedial treatment of diseased teeth, and their connections, has now reached a point, which is a surprise to the most distinguished professors of general medicine and surgery. They find the most recondite principles of the parent science, and the nicest skill in treatment in familiar use in our improved practice; and they are witnesses that the same talent, perseverance and devotion are employed upon the teeth, that the oculist, the aurist and lithotomist require in their older and better appreciated surgery. The claim to equally liberal remuneration is, therefore, well-founded in the equal character of our professional qualifications, and the equal value of our operations: but vocations are no longer merely mechanical, but strictly professional, and entitled to all the rank and rights thence resulting.

*Third.*—Every practitioner of eminent competency knows that the required attainments are only to be had through such study, observation, training and practice as must have for their inducements and reward handsomely liberal emoluments, or the profession can neither enlist or develop the requisite abilities within its ranks. Talent sure of higher consideration and better recompense elsewhere, instinctively avoids a beggarly and depreciated occupation. A business must pay the highest remuneration in respect and profit, if it would secure the highest qualifications in its membership. Modesty and moderation in some things is commendable, because they hit the mean between extremes, but in some other things they are far more likely to land in the meanest of the lowest of extremes. Liberality in compensation, taking human nature as it is, and human things as we find them, is the only means of improvement in practice, and progress in knowledge of a growing profession. Let the men who are in a position to answer our highest expectations establish such ample rates for their best work, that they, and those who are to come to them, may be able to indulge their ambition for superiority; and cheap wages and cheap work, whether banished or not, will cease to be a reproach to our profession and an impediment to its advancement.

We want men of genius more than we want any thing else, and the public both wants and knows this want also, and will gladly stand the tariff of protection which will encourage their production. The laws of human nature remaining the same, niggardly wages must be the corresponding meanness of desert. A man has but little critical ability in dentistry, who cannot distinguish a dollar plug from a five dollar filling by examining it in the patient's tooth, without further scrutiny, and we all know that he is either a very poor man or a very poor economist who chooses the cheaper article of the two. There are too many nice points in the operation, and too many drafts upon the time and patience of the operator, to put him with safety to the patient upon two or three dollars a day, even with roast beef added, which he will not have the pleasure to enjoy or the vigor to digest. For the sake of the profession's advancement, and the patient's real interest, it is well to compensate five times the skill and care which the lowest rates will any way allow. Let it be understood here distinctly that I am not censuring, much less despising, the more moderate rates of charging, which are also more general. So long as they are kept any where within the limits of respectability, and the work done under them does nothing specially to discredit the profession.

There is a large amount of dental practice which demands only middling merchantable sort of work, and it would be vain, if not unjust, to deny it. The question in that quarter lies between the patient and the practitioner, and they must settle it. Neither party is responsible to the progressive wing of the profession. I am only insisting that there shall be such a wing, and offering the argument for it which presents itself. Our profession, like those of law, medicine and divinity, has room for considerable variety of ability, and even demands considerable accommodation to its varied objects, so that we might be divided into several groups without at all severing the fraternal unity. Let there be no unwarrantable envy, and no revenging scorn amongst us. The moderate party suffer nothing in business by the competition of the more exacting, and they, in their turn, should not complain on their own account. While they can maintain their own position, however, for highest and unselfish reasons they could wish it were otherwise throughout the brotherhood. There is no danger that the less ambitious practitioners will be tempted to pretensions in the matter of fees above the value of their work, the imposture is too open to observation for that; and, what is still better, the higher price would most generally tempt to better deserving by compensating it, and so the right is tolerably well secured and the wrong prevented, and, on the other hand, there is fortunately enough of strength in the faculty, and enough of its proper enthusiasm among the aristocrats of price, to sustain the aristocracy a talent if they have it, if they have not, the descent is alike easy and inevitable, for, life and business have their natural laws which will be obeyed where they are resisted. The only evil is in not discovering them soon enough, or understanding them well enough to get their greatest benefits.

There is yet another point which must not be overlooked. I have spoken of liberal compensation as a *condition* of professional advancement, a *right* of the meritorious practitioner, a duty and interest of the patient's, and a point of *honor* to the profession. These all bear upon the pecuniary reward of valuable services, and this is open to impeachment by that spirit of selfishness in criticism which has the knack of finding its own ugly features most prominent in every bright object which it gazes at; but even this money claim, ministers to another motive and higher style of reward than envy and suspicion can touch with depreciation. I allude to the leisure and the opportunities which ample resources afford for the generous employment of professional skill, in cases of worthy persons who can afford us only the pleasure and the pride of professional munificence, in compensation for the benefits bestowed.

There are men in the fraternity who would feel the restraint of circumstances in this respect, as sharply at least as in the meagreness of income which compelled it. No profession is honorable, or dignified, or happy that does not provide for the very many instances in which the finest feelings of our nature ask to be indulged in liberal deeds. Indeed, there are not a few instances where only medium charges can be made with comfort to the patient, where yet extraordinary pains and skill, and time are taxed to meet the necessities of the case, and gratify the operator's pride of excellence in his work. A tariff of prices that makes no room, and provides no possibility for occasions which so frequently present themselves in every good practice must drive every gentleman from the fraternity, or sink him in his business below the standard that he would proudly prescribe for himself as a man.

